# Identification of the Adapter Molecule MTSS1 as a Potential Oncogene-Specific Tumor Suppressor in Acute Myeloid Leukemia

**DOI:** 10.1371/journal.pone.0125783

**Published:** 2015-05-21

**Authors:** Mirle Schemionek, Behzad Kharabi Masouleh, Yvonne Klaile, Utz Krug, Katja Hebestreit, Claudia Schubert, Martin Dugas, Thomas Büchner, Bernhard Wörmann, Wolfgang Hiddemann, Wolfgang E. Berdel, Tim H. Brümmendorf, Carsten Müller-Tidow, Steffen Koschmieder

**Affiliations:** 1 Department of Hematology, Oncology, Hemostaseology, and Stem Cell Transplantation, Faculty of Medicine, RWTH Aachen University, Aachen, Germany; 2 Department of Urology, University of Muenster, Muenster, Germany; 3 Department of Medicine A, Hematology, Oncology, Pneumology, University of Muenster, Muenster, Germany; 4 Institute for Medical Informatics, University of Muenster, Muenster, Germany; 5 Department of Internal Medicine III, University of Munich, Munich, Germany; 6 Membership of the German Society of Hematology and Oncology (DGHO), Berlin, Germany; 7 Clinical Cooperation Group Acute Myeloid Leukemia, Helmholtz Zentrum Munich, German Research Center for Environmental Health, Munich, Germany; Emory University, UNITED STATES

## Abstract

The adapter protein metastasis suppressor 1 (MTSS1) is implicated as a tumor suppressor or tumor promoter, depending on the type of solid cancer. Here, we identified Mtss1 expression to be increased in AML subsets with favorable outcome, while suppressed in high risk AML patients. High expression of MTSS1 predicted better clinical outcome of patients with normal-karyotype AML. Mechanistically, MTSS1 expression was negatively regulated by FLT3-ITD signaling but enhanced by the AML1-ETO fusion protein. DNMT3B, a negative regulator of MTSS1, showed strong binding to the MTSS1 promoter in PML-RARA positive but not AML1-ETO positive cells, suggesting that AML1-ETO leads to derepression of MTSS1. Pharmacological treatment of AML cell lines carrying the FLT3-ITD mutation with the specific FLT3 inhibitor PKC-412 caused upregulation of MTSS1. Moreover, treatment of acute promyelocytic cells (APL) with all-trans retinoic acid (ATRA) increased MTSS1 mRNA levels. Taken together, our findings suggest that MTSS1 might have a context-dependent function and could act as a tumor suppressor, which is pharmacologically targetable in AML patients.

## Introduction

Acute myeloid leukemia (AML) comprises a heterogeneous group of malignancies that are characterized by a maturational block of myeloid cell development and can be classified according to their distinct phenotypes and / or oncogenes. Although therapeutic interventions in AML have steadily improved, the implementation of disease specific treatment strategies is still inapplicable for most AML entities. Moreover, acquisition of novel mutations in addition to insufficient disease eradication of malignant self-renewing cells may result in subsequent relapse. In order to achieve better control of AML stem cells and improve long-term survival of patients a better understanding of the pathogenesis in various AML subtypes is required [1]. Different AML subtypes are a result of unique mutations, such as FLT3-ITD conferring resistance to conventional therapy and correlate with poor clinical outcome [2]. In comparison, other mutations such as the translocations t(8;21) or t(15;17) resulting in the AML1-ETO or PML-RARα fusion molecules, respectively, are associated with a lower risk and far better prognosis [3].

Therefore, the identification of potential tumor suppressors or oncogenes which may be either suppressed or activated by FLT3-ITD signaling in direct contrast to AML1-ETO patients could help to improve our understanding of the unique signaling patterns. The identification of such molecules would help in the development of more personalized and successful therapeutic approaches.

Recently, we have described genetic mutations and epigenetic alterations (“epimutations”) in the gene encoding for DNA methyltransferase 3A (DNMT3A) [4] which affect DNMT3A isoform expression and are associated with an inferior prognosis in AML, clustering together with FLT3-ITD mutations but not with AML1-ETO translocations. Overexpression of DNMT3B, a related gene, was shown to confer a poor prognosis in AML [5]. However, the mechanism of how DNMT3B affects AML cells is currently unknown. Interestingly, the adapter molecule and potential tumor suppressor molecule Metastasis Suppressor 1 (MTSS1) has recently been described to be a transcriptional target of DNMT3B in hepatocellular cancer [6]. Moreover, the authors demonstrated that the multi domain adapter molecule MTSS1 functions as a tumor suppressor *in vivo*. MTSS1 has previously been shown to be downregulated in metastases of a variety of solid tumors. Moreover, low MTSS1 expression levels confer a poorer prognosis in breast and ovarian cancer and higher expression levels correlate with improved overall survival rates [7,8]. The prognostic value of MTSS1 expression has also been demonstrated for esophageal squamous cell carcinoma in which patients with high MTSS1 expression levels had a favorable prognosis compared to those who had reduced MTSS1 expression levels [9]. However, in colorectal cancer high MTSS1 expression has recently been shown to correlate with poor prognosis [10] and to be associated with disease progression in a subset of human melanomas [11]. The exact role of this adaptor molecule that links intracellular signaling pathways with actin remodeling [7]^;^[12]^;^[13] is still under debate and may likely depend on the cell type and molecular context. Recent genetic knockout studies for MTSS1 suggest an important role in the hematopoietic system. Specifically, MTSS1 knockout mice have an increased propensity to develop aggressive B cell lymphomas [14]. This suggests that MTSS1 has a tumor suppressing function in hematopoietic malignancies. However, whether MTSS1 plays a role in the pathogenesis of myeloid neoplasms and how MTSS1 expression and MTSS1-mediated signaling are altered in the context of specific hematopoietic oncogenes remains unclear.

Here, we provide evidence that MTSS1 expression is differently expressed in specific AML subsets. Interestingly, low MTSS1 expression correlated with decreased overall survival in normal-karyotype AML, possibly involving DNMT3B overexpression. Moreover, Mtss1 downregulation may be an indicator or involved in the pathogenesis of unfavorable AML subtypes, as MTSS1 expression was low in FLT3-ITD but high in AML1-ETO associated AML. Finally, the expression of MTSS1 could be reverted by pharmacological inhibition which specifically targets either the PML-RARα or FLT3-ITD oncogenes leading to increased expression of MTSS1.

## Materials and Methods

### Primary human samples and human cell lines

The human and murine cell lines were obtained from DSMZ, Braunschweig, Germany. We maintained human leukemia cells (U937, K562, NB4, Kasumi-1 and MV4-11, in Roswell Park Memorial Institute medium (RPMI-1640, Invitrogen, Carlsbad, CA) with GlutaMAX containing 10% fetal bovine serum, 100 IU ml^-1^ penicillin and 100 μg ml^-1^ streptomycin at 37°C in a humidified incubator with 5% CO_2_. The murine 32D cell lines were cultured in RPMI with L-glutamine and 10% FCS; 32D cultures were supplemented with murine IL-3 obtained from supernatant of WEHI-3B cells. Stably transfected U937 cell lines expressing in a Zn^2+^-inducible fashion PML-RARA or empty vector as control have been previously described [15]. Written consent was obtained from all patients before sample analysis, and the study was approved by the ethics committee of the Faculty of Medicine, Wilhelms-University of Muenster.

### Quantitative RT-PCR and genomic PCR

RNA was isolated using RNAeasy kit (QIAGEN) according to the manufacturer's instructions. RNA (1 μg) was reverse transcribed using oligo-d(T) primer and random hexamers. cDNA was generated with M-MLV reverse transcriptase (Promega) for 1 hour at 42°C. The quantitative reverse-transcribed polymerase chain reaction (qRT-PCR) for gene expression was performed using either a combination of Primer and 6-carboxyfluorescein (FAM)-labeled Probe or SYBER Green. Commercially available TaqMan assays (Applied Biosystems by life Technologies) were used for human (HS00207341_m1) or murine MTSS1 (Mm00460614_m1) expression analysis. For relative quantification we included detection of GAPDH as a housekeeping gene and calculated the expression of our gene of interest as percent of GAPDH.

### Western blotting

After harvest, 2x10^6^ cells were washed twice with PBS and lysed in CelLytic MT buffer (Sigma-Aldrich) supplemented with Mini Complete protease inhibitor (Roche), 1% phosphatase inhibitor cocktail (Calbiochem), and 1mM PMSF. After 10 min incubation on ice and centrifugation at 11,000g for 10 min at 4°C, the protein concentration was determined by BCA Assay (Thermo Scientific). Protein samples were loaded on 10% Bis-Tris gels and transferred on a PVDF membrane (BioRad). For protein detection of MTSS1 or GAPDH primary antibodies from Cell Signaling (#4386, clone N747, 1:1000 dilution) or Santa Cruz (sc-32233, clone 6C5, 1:2000 dilution) were used followed by a secondary goat anti-rabbit HRP conjugated antibody (Dako, 1:10000) and light emission analysis using Fusion SL system (PeqLab).

### Pharmacological inhibitor and differentiation studies

0.5μM All-*trans* retinoic acid (Stock 10μM in DMSO; Sigma-Aldrich) was added as differentiation agent to NB4, U937 PMT control and U937 PMT RARα cells. Control samples were incubated using equivalent volume of DMSO vehicle. Cells were harvested at the indicated time points and subjected to quantitative RT-PCR or FACS analysis. PKC-412 was similarly dissolved in DMSO and cells were treated at indicated time points.

### Flow cytometry

For flow cytometry analysis 2x10^5^ cells were harvested and washed using phosphate buffered saline (PBS) including 2% FCS. Cells were resuspended in 100 μL PBS/2%FCS, and incubated with either PE-labeled anti-CD11b (clone M1/7015.1, Cymbus Biotechnology) or PE-labeledanti-CD11c (clone B-ly6, BD Biosciences) antibody for 10 minutes on ice for staining of granulocytes or macrophages. For detection of unspecific binding cells were incubated with PE-labeled Mouse IgG1-control antibody. All antibodies were used in a 1:100 dilution. Cells were washed, resuspended in 400μl PBS/2%FCS and analyzed by flow cytometry (FACSCalibur, BD Biosciences). We therefore first gated on living cells via SSC/FSC profile and subsequently analyzed surface expression of the respective antigen using 1x10^4^ gated viable cells.

### Renilla / Firefly Reporter System

The Dual-Luciferase Reporter Assay System (Promega) was used to measure luciferase activity of cells transfected with indicated MTSS1 constructs using a Synergy2TM luminometer (BioTek, Winooski, VT). Sample preparation was performed according to the manufactures protocol. Luciferase activities are shown as a Firefly/Renilla ratio.

### Chromatin immunoprecipitation (ChiP)

ChIP experiments were performed using the Active Motif ChIP-IT Express Chromatin Immunoprecipitation Kit. 5x10^6^ NB4 or Kasumi-1 cells were fixed with formaldehyde for 10 minutes, lysed and DNA was sheared using a Bioruptor Pico (Diagenode) for 12 cycles (30 sec on, 30 sec off). Immunoprecipitation was conducted with a DNMT3B antibody (Santa Cruz; H-230) or an IgG antibody (Abcam) as control to test for unspecific binding. Interaction between DNMT3B protein and MTSS1 promoter was analyzed by quantifying precipitated ChIP-DNA using real-time PCR and SYBR Green. Percent of bound DNA was calculated and compared to an input control. (2^-(Ct (IP) − Ct (Input))^). Primers used for amplification of the precipitated DNA fragments were published previously [6].

### Patient outcome and gene expression microarray data

Gene expression microarray and patient outcome data were obtained from the GEO database accession numbers GSE1159 [16], GSE15646 [17], GSE8023[18] and GSE12417[19]. Data was processed with Cluster and visualized by Treeview software 1.6. Expression level of a gene in a sample was determined by the average of expression values from multiple probe sets on the array representing this gene.

### Statistical analysis

Statistical analyses were performed using Student t test (normal distribution) or Mann-Whitney U test (when normal distribution was not given). P less than .05 was considered as indicating statistically significant differences.

## Results

### MTSS1 is overexpressed in AML1-ETO^+^ but downregulated in FLT3-ITD^+^ AML

**Fig 1:**
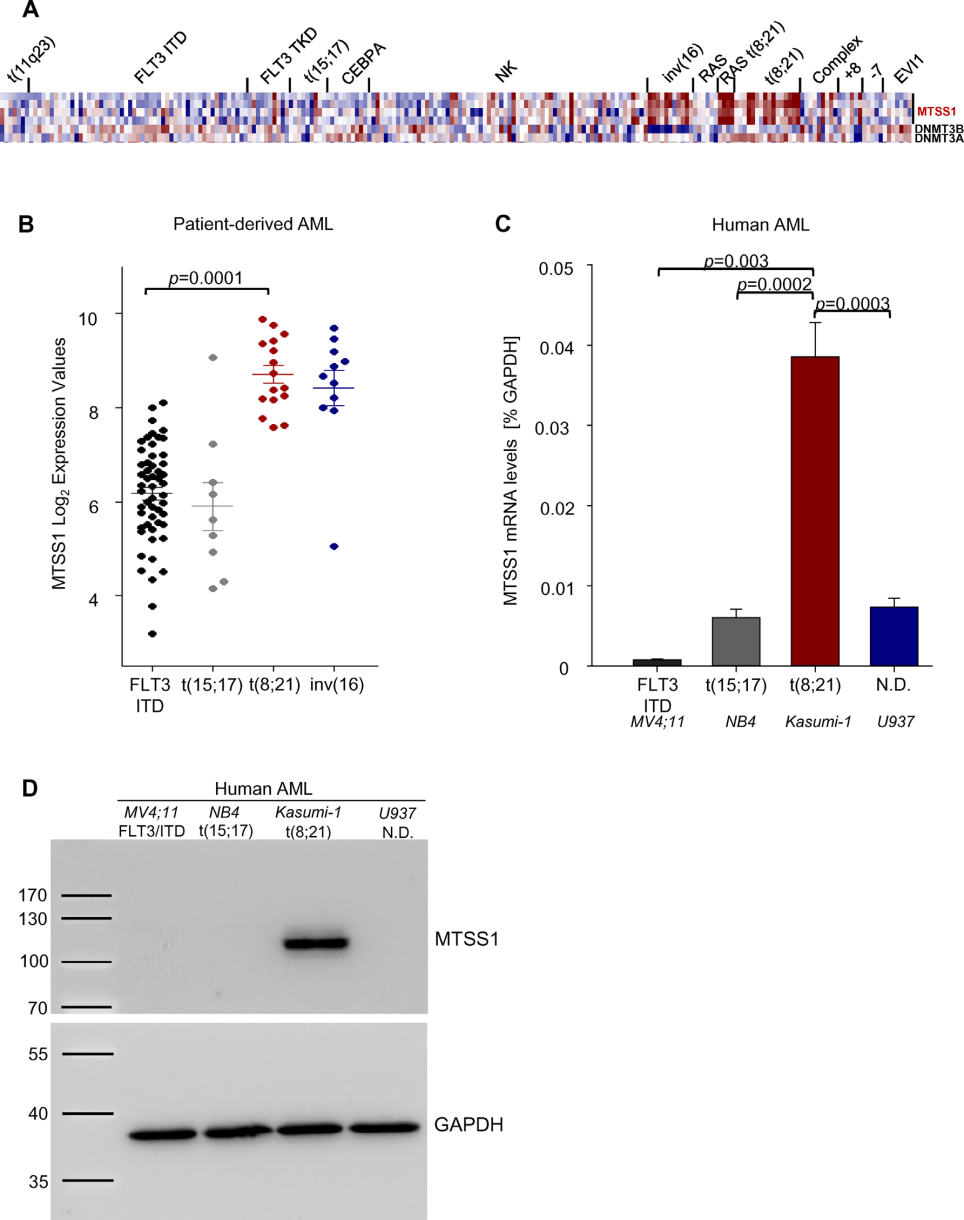
MTSS1 is upregulated in t(8;21) and inv(16) AML patients. The gene expression profiling of MTSS1, DNMT3B and DNMT3A in different AML subsets (GEO accession number GSE1159) is shown (**A** and **B**). MTSS1 mRNA levels were measured by qRT-PCR in human AML cell lines carrying either a FLT3-ITD mutation (MV4-11), t(15;17) (NB4), t(8;21) (Kasumi-1) or undefined genetic changes (U937) in relative expression to GAPDH (n = 3; **C**) which was verified at the protein level by Western Blot analysis using GAP-DH as a loading control (**D**).

The prognostic value of MTSS1 downregulation has been demonstrated for solid tumors such as breast and prostate cancer. Moreover, functional data have revealed a tumor suppressing function also in hematopoietic malignancies. To study the function of MTSS1 in AML subsets we used publicly available gene expression data [16] and analyzed the expression of DNMT3A, DNMT3B, and MTSS1 in different AML subsets. We found that MTSS1 was strikingly overexpressed in patients carrying the favorable t(8;21) and inv(16) translocations compared to patients with FLT3-ITD mutation correlating with poor prognosis as well as t(15;17) (Fig 1A and 1B). MTSS1 expression was not altered in the cluster with RAS mutations only, but was increased in the cluster of patients which harbored both RAS mutations and a t(8;21) translocation, suggesting a link between the AML1-ETO fusion molecule and MTSS1 (Fig 1A). Further proof that MTSS1 expression is differentially regulated by oncogenic alterations in AML cells came from the fact that MTSS1 mRNA levels were significantly higher in an AML cell line carrying the t(8;21) translocation (Kasumi-1) than in FLT3-ITD positive cells (MV4-11), PML-RARα t(15;17) positive cells (NB4) or U937 cells (Fig 1C). These effects on MTSS1 RNA translated into significant differences in MTSS1 protein levels in AML1-ETO positive vs. FLT3-ITD and PML-RARα positive cell lines, as detected by Western blotting (Fig 1D).

### MTSS1 expression correlates with improved survival in normal karyotype AML

**Fig 2:**
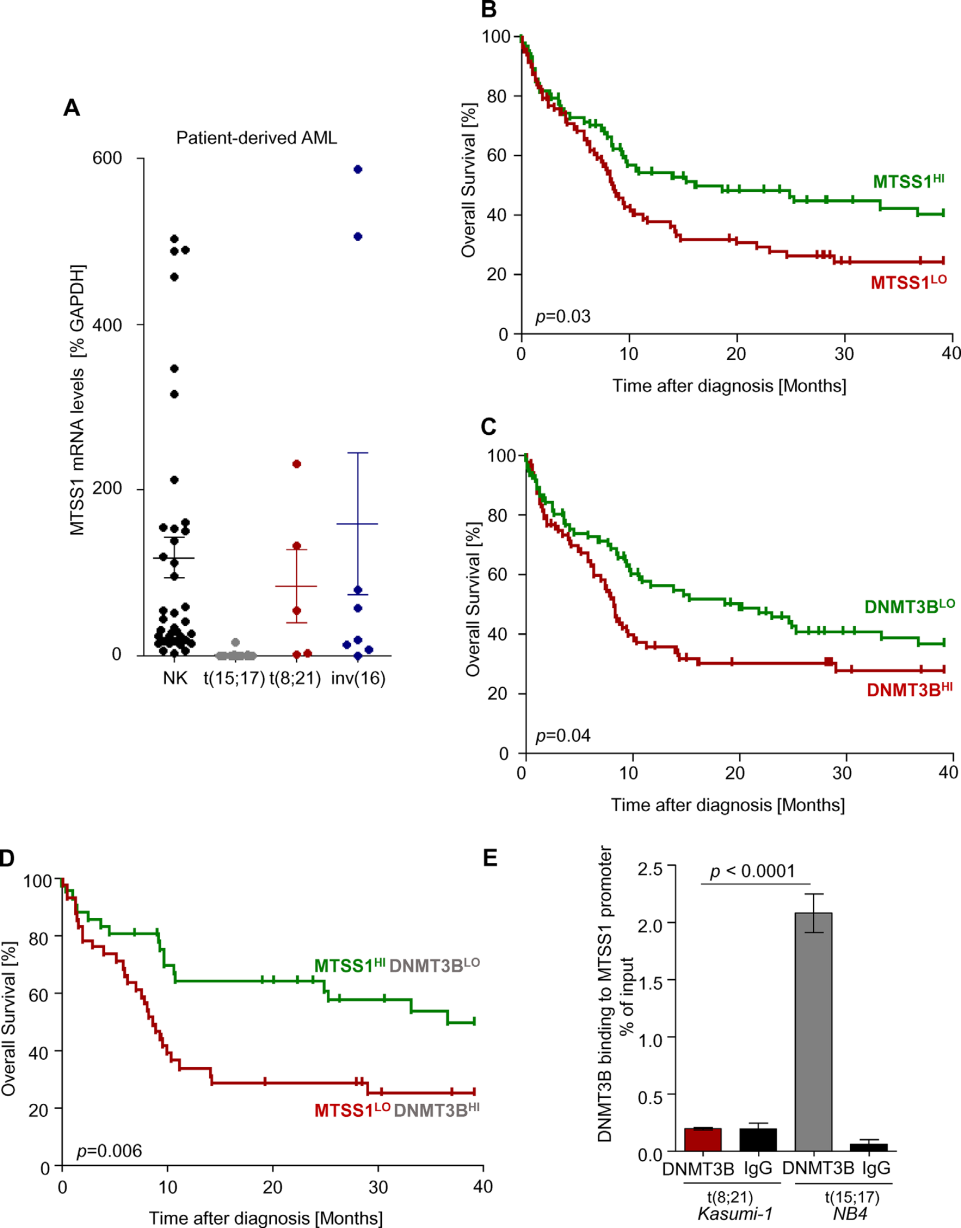
MTSS1 expression correlates with better clinical outcome of normal karyotype-AML patients. MTSS1 mRNA levels were measured in patient-derived AML samples (n = 66) with either a Normal Karyotype (NK, n = 38), t(15;17) (n = 15), t(8;21) (n = 5) or inv(16) (n = 8) aberration by qRT-PCR and are expressed as % of GAPDH (**A**). Using the Leukemia Gene Atlas [37] and data from the German AMLCG 1999 clinical trial [19], two groups of patients (above vs. below the median expression of MTSS1) were analyzed for overall survival (OS) in NK AML patients (n = 163, logrank test P = 0.03; GEO accession number GSE12417; **B**). Similarly, in the same trial, patients with a DNMT3B expression above vs. below the median were analyzed for survival (n = 163, logrank test P = 0.04; **C**). We then segregated patients into two groups based on their MTSS1 and DNMT3B expression levels according to high MTSS1 and low DNMT3B or low MTSS1 and high DNMT3B expression and assessed overall survival (AMLCG 1999, n = 163, logrank test P = 0.006; **D**). Binding of DNMT3B to the MTSS1 promoter was analyzed by chromatin immunoprecipitation experiments using NB4 and Kasumi-1 cells. Precipitated ChIP-DNA was quantified using real-time PCR and SYBR Green for MTSS1 promoter region -864/-645 **(E)**.

In our cohort of patients with AML, MTSS1 expression was confirmed by qRT-PCR to be increased in t(8;21) or inv(16) AML samples as compared to t(15;17) (Fig 2A). Interestingly, when we analyzed samples from patients with normal karyotype AML, we found MTSS1 expression to be highly variable (Fig 2A). We reasoned that since high MTSS1 expression was found in prognostically favorable AML subgroups, the prognosis of patients with normal karyotypes may differ according to their level of *MTSS1* expression. Indeed, when 163 patients with normal karyotype-AML in the German AMLCG 1999 clinical trial [19] were separated into two groups depending on whether their expression of MTSS1 is below or above the MTSS1-probeset, median across all samples, patients with high expression levels of *MTSS1* (*MTSS1* ^HI^) showed a significantly improved overall survival suggesting that MTSS1 is a predictor of favorable outcome (Fig 2B). In the same clinical trial, high expression of *DNMT3B*, which has been shown to impede MTSS1 expression, showed reversed patterns and correlated with decreased overall survival (Fig 2C). To study if there is a link between MTSS1 and DNMT3B, we segregated the patients in two groups based either on their high MTSS1 and low DNMT3B (MTSS1^HI^—DNMT3B^LO^) or low MTSS1 and high DNMT3B expression (MTSS1^LO^—DNMT3B^HI^). Strikingly, patients with high MTSS1 and low DNMT3B expression showed a highly significant increased overall survival rate compared to those with low MTSS1 and high DNMT3B expression (Fig 2D). DNMT3B binding to the MTSS1 promoter has previously been described to occur at -864/-645 bp upstream of the transcriptional start site and acts as a mechanism that allows for transcriptional suppression of MTSS1 in hepatocellular carcinoma [6]. To analyze for a direct binding of DNMT3B to the respective MTSS1 promoter region in AML, we performed chromatin immunoprecipitation experiments. We previously detected increased MTSS1 expression in t(8;21) positive cells but not in t(15;17) positive AML (Fig 1B and 1C) and therefore compared DNMT3B binding to the MTSS1 promoter using these two cell types (Fig 2E). We confirmed binding of DNMT3B to the MTSS1 promoter region (-864/-645) in t(15;17) positive cells. Interestingly, this interaction was not detectable in t(8;21) MTSS1 highly expressing AML cells. These data confirm a direct link between DNMT3B activity and decreased MTSS1 expression also in a subset of AML.

### MTSS1 expression is upregulated after ATRA-mediated differentiation in t(15;17) AML

**Fig 3:**
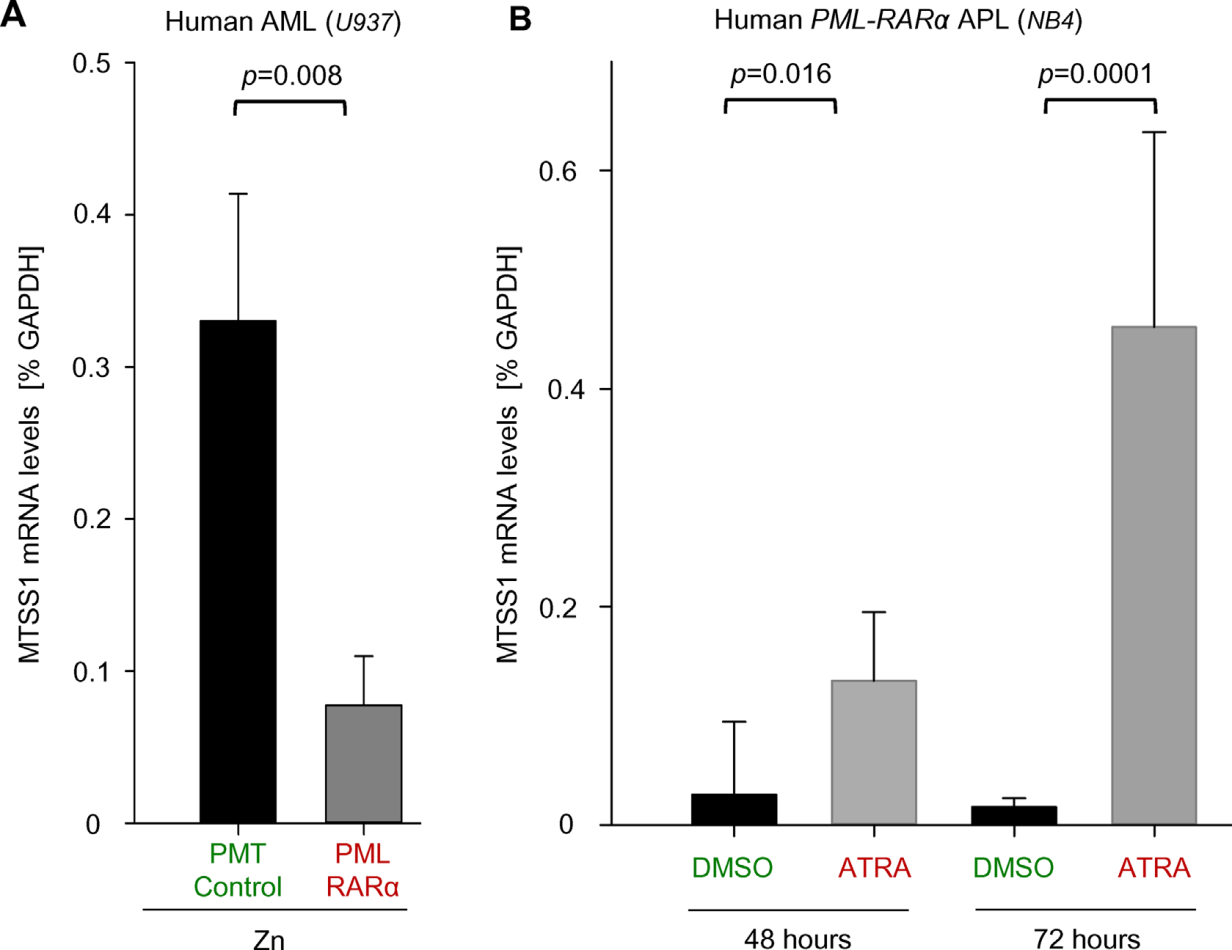
MTSS1 expression is increased by ATRA treatment in t(15;17) AML. MTSS1 mRNA levels were measured in human AML cells (U937) transduced with either empty vector control (PMT Control) or an inducible PML-RARα (PR9) after 24 hours of activation with zinc (Zn) by qRT-PCR (n = 3; **A**). Similarly, MTSS1 mRNA levels were measured after treatment with all-trans retinoic acid (0.5μM ATRA) or 0.5μM DMSO control after 48 and 72 hours in human AML cells carrying the PML-RARα translocation (NB4) (n = 3; **B**).

The t(15;17) translocation is only found in the acute promyelocytic leukemia (APL) subset of AML and results in the PML-RARα fusion molecule. The oncogenic protein induces a differentiation block that can be reversed by treatment with all-trans retinoic acid (ATRA) [20]. Our findings suggest that MTSS1 is downregulated in APL patients (Figs 1B and 2A). To study if MTSS1 expression is affected by the presence of PML-RARA, we used a zinc-inducible system of stable PML-RARα overexpression in human AML cells (U937, PR9), which usually do not carry the PML-RARα fusion molecule. Upon induction of ectopic PML-RARα expression by the addition of zinc to the cell cultures, MTSS1 mRNA levels significantly decreased in PML-RARα expressing but not control cells (U937, PMT empty vector), suggesting a direct link and potential tumor suppressive function of MTSS1 in PML-RARα positive cells (Fig 3A). We next studied if MTSS1 mRNA levels could be rescued by treatment with ATRA. Treatment with ATRA both in these stably transfected PML-RARα expressing U937 cells as well as in t(15;17)-carrying human AML cells (NB4) led to differentiation as verified by increased expression of myeloid markers such as CD11b and CD11c after 72 hours of treatment (S1 Fig). More importantly, when NB4 cells were treated with ATRA, MTSS1 mRNA levels were significantly increased 2.9-fold after 48 hours and progressed after 72 hours of treatment to 7.8-fold (Fig 3B). In line with these data, there was a trend of increasing Mtss1 levels (1.9-fold; p = 0.05) in U937 PR9 cells after 72 hours of treatment but not in empty vector U937 control cells (S2 Fig). These findings suggest that MTSS1 expression is downregulated by PML-RARα and that this can be reverted by ATRA also in patients with APL.

### MTSS1 expression is positively regulated by AML1-ETO in AML patients

**Fig 4:**
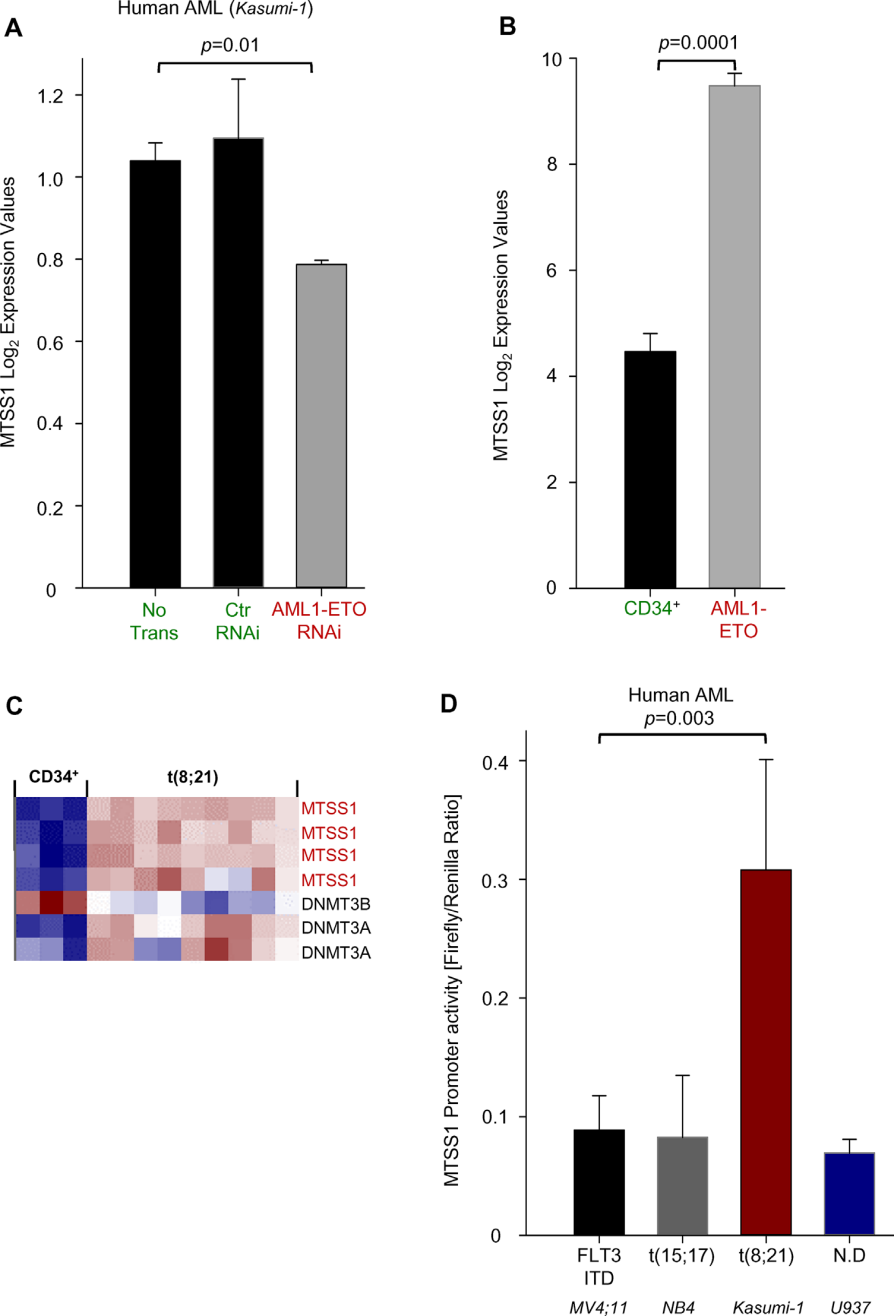
MTSS1 is positively regulated by AML1-ETO in human AML cells. The gene expression of MTSS1 in human AML cells (Kasumi-1 without transduction (no Trans), scramble siRNA (ctr RNAi) or siRNA targeting the AML1-ETO translocation is shown (GEO accession numbers GSE15646; **A**). Similarly, the gene expression of MTSS1, DNMT3B and 3A are measured in a gene expression analysis of human cord-blood derived CD34^+^ cells and patient-derived AML samples with the AML-1 ETO translocation (GEO accession numbers GSE8023; **B** and **C**). Human AML cell lines carrying either a FLT3-ITD mutation (MV4-11), t(15;17) (NB4), t(8;21) (Kasumi-1) or not defined (U937) were transduced with a MTSS1 firefly / renilla construct and the MTSS1 promoter activity as a normalized relative ratio was measured (n = 3;**D**).

Since MTSS1 expression was high in t(8;21) positive cell lines and primary AML samples, we next studied whether there was a direct link between MTSS1 and AML1-ETO. Using publicly available databases, we could show that there is a trend of MTSS1 reduction after siRNA-mediated knockdown of AML1-ETO in human t(8;21) positive AML cells (Kasumi-1) (Fig 4A). Similarly, in patient-derived AML1-ETO positive primary AML cells, MTSS1 mRNA levels were significantly increased compared to human cord-blood-derived CD34^+^ myeloid cells (Fig 4B). When assayed for expression, Mtss1 was low (blue squares) in CD34 positive normal cells but increased in t(8;21) cells (red squares) and this was also true for DNMT3A expression. Reversely, expression of DNMT3B was high (red squares) in CD34 positive normal cells but low in t(8;21) cells (blue and white squares (Fig 4C). Using our own experiments, we next tested the hypothesis if MTSS1 promoter activity is directly regulated by AML1-ETO, using the same Kasumi-1 cell line that had suggested a trend of AML1-ETO siRNA mediated downregulation of MTSS1 (Fig 4A). Luciferase promoter assay using a promoter region containing a fragment of -276 to -13 bp has previously been described to confer highest Mtss1 expression. The indicated nucleotide position -276 to -13bp is related to the 5´end of annotated Mtss1 mRNA sequence with the accession number (NM_014751.5) [21]. Our data demonstrate that MTSS1 promoter activity was significantly higher in human t(8;21) positive AML cells (Kasumi-1) when compared to various AML1-ETO negative cell lines (Fig 4D). This suggests that the AML1-ETO fusion molecule is a positive regulator of MTSS1.

### Pharmacological inhibition of FLT3-ITD causes upregulation of MTSS1 expression

**Fig 5:**
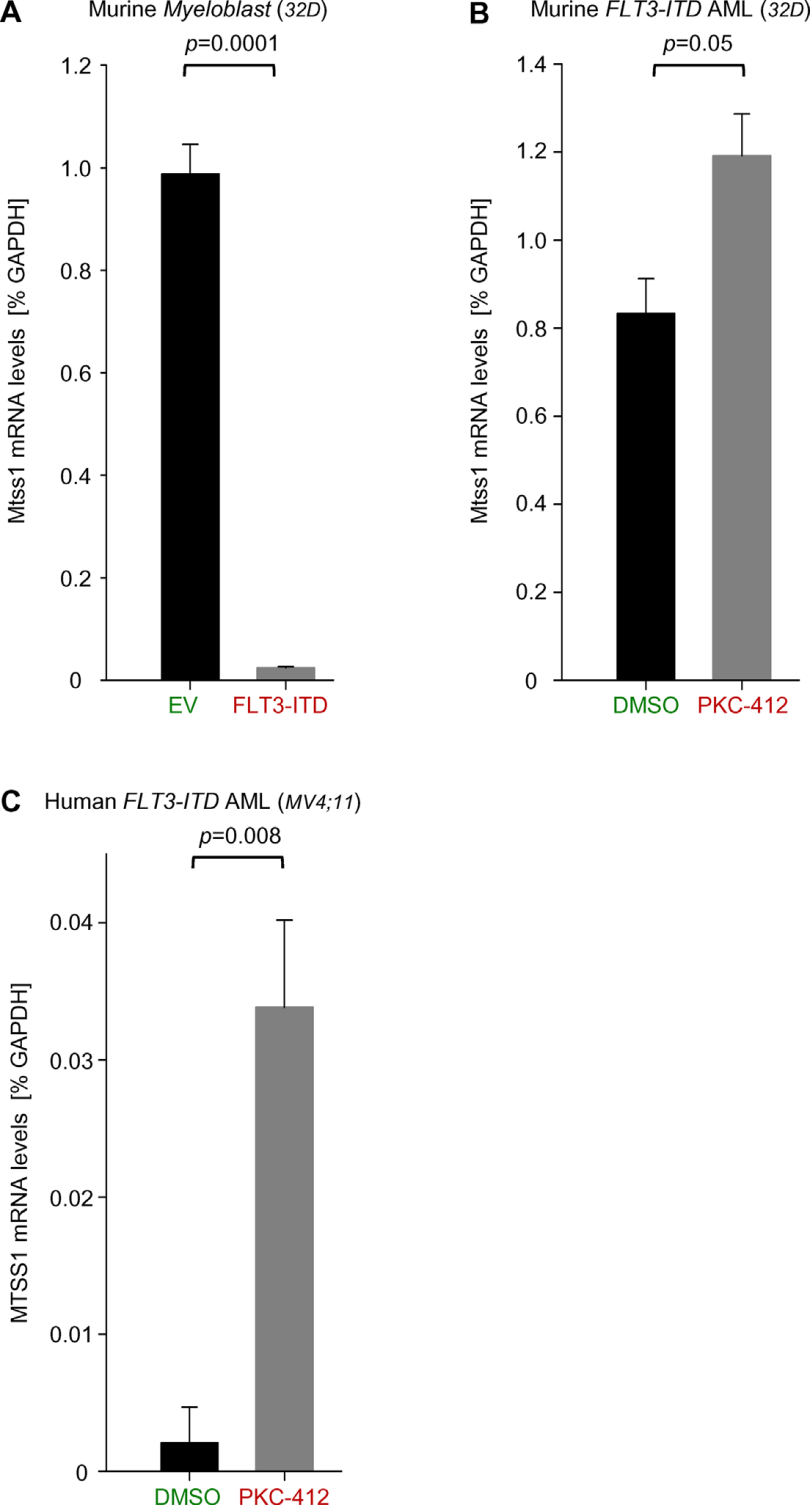
FLT3-ITD mediated suppression of MTSS1 is pharmacologically reverted by PKC-412. *Mtss1* mRNA levels were measured in murine 32D cells transduced with either empty vector control (EV) or a FLT3-ITD overexpression vector by qRT-PCR (FLT3-ITD; n = 3; **A**). Similarly, FLT3-ITD transduced murine 32D cells and human FLT3-ITD^+^ AML cells (MV4;11) were treated with the pharmacological FLT3-ITD inhibitor PKC-412 and DMSO control and *MTSS1* mRNA levels were measured after 24 hours (n = 3; **B** and **C**).

Finally, since MTSS1 expression was decreased in FLT3-ITD AML patient samples (Fig 1A), we next studied if there is a potential novel link between MTSS1 and FLT3-ITD mutations. Strikingly, in a murine AML cell line model of 32D cells that were retrovirally transduced with a FLT3-ITD overexpressing vector, mRNA levels of MTSS1 were significantly downregulated 30-fold, suggesting that MTSS1 is negatively regulated by FLT3-ITD signaling (Fig 5A). We next assessed if this downregulation could be reversed using a pharmacological FLT3-ITD inhibitor midostaurin (PKC412) which is currently being tested in clinical trials of Flt-ITD positive AML [22]. Treatment of FLT3-ITD transduced murine 32D cells showed a trend for an upregulation (p = 0.05) of MTSS1 mRNA levels (Fig 5B). To exclude the possibility that the potential of PKC412 to reverse MTSS1 downregulation was underestimated due to the high levels of FLT3-ITD in the retrovirally transduced cells, we tested PKC412 in human AML cells carrying a genetic FLT3-ITD mutation (MV4-11). Pharmacological inhibition of FLT3-ITD significantly increased MTSS1 mRNA levels approximately 12-fold suggesting that downregulation of MTSS1 expression by FLT3-ITD is kinase-dependent (Fig 5C). As expected, ATRA was not able to increase MTSS1 levels in FLT3-ITD positive cells (S3 Fig), suggesting that the effects in NB4 and MV4-11 cells were truly oncogene-related.

## Discussion

Here, we found that MTSS1 expression was high in AML subsets with good prognosis (t(8;21), inv(16)), and a fraction of NK-AML, but was suppressed in AML subsets with poor outcome (FLT3-ITD). While earlier studies of MTSS1 had mainly focused on solid cancers and lymphomas, this is the first study in myeloid neoplasms providing evidence for an important function of MTSS1 as a potential tumor suppressor in AML and as a predictor of clinical outcome.

MTSS1 (also termed MIM for “missing in metastasis”) had initially been identified as a gene that was downregulated in cell lines from metastatic bladder, prostate, and breast cancer as compared to cell lines from patients with non-metastatic malignancies [23]. This observation was subsequently confirmed in metastatic specimen from patients with-among others- gastric cancer [24], hepatocellular carcinoma [6], breast cancer [7], bladder [21] and prostate cancer [25]. Several mechanisms for MTSS1 downregulation have been proposed including DNA methylation [21], transcriptional suppression by DNMT3B [6] or microRNA-mediated effects [26–31]. Several groups have shown that lack of MTSS1 promotes tumor growth through enhanced cell migration and invasion as well as enhanced cell proliferation [6,7,25,30,32]. However, the exact role of Mtss1 might depend on the cell context and disease type as several reports have recently reported a possible tumor-promoting function [10,11].

Mice with targeted disruption of MTSS1 (MTSS1 knockout mice) show altered cell polarity, motility, receptor signaling, and membrane ruffling [14], altered cytoskeleton organization and cell-cell junctions [33], and they ultimately develop aggressive B cell lymphomas [14]. The mechanism of lymphomagenesis possibly involved aberrant interactions between B lineage cells and the lymphoid microenvironment as well as a differentiation block within the B lymphoid compartment. Furthermore, MTSS1 was found to be downregulated in cells from patients with B cell lymphomas and pre-B-ALL [14]. Thus, the data from solid tumors suggest that MTSS1 may not only be a prognostic AML biomarker but that it may in fact act as an oncogene-specific tumor suppressor in AML.

The opposing patterns of DNMT3B and MTSS1, the fact that DNMT3B overexpression is associated with poor prognosis, and the demonstration that DNMT3B binds directly to the 5´-flanking region of the MTSS1 promoter to downregulate MTSS1 expression [6] implicate MTSS1 as one of the major downstream factors of DNMT3B which may at least in part account for the poor prognosis of DNMT3B-overexpressing AML subsets. It was tempting to speculate that DNMT3B may be downregulated in AML1-ETO positive cells, and indeed we found this to be the case (Fig 4C). Functionally, we showed binding of DNMT3B to the MTSS1 promoter in PML-RARα positive cells while DNMT3B binding to the promoter was absent in AML1-ETO cells (Fig 4E), further supporting the idea that MTSS1 is released from DNMT3B-mediated repression by AML1-ETO. Future experiments will need to address whether AML1-ETO directly inhibited DNMT3B expression to allow for high MTSS1 expression. Of course, we cannot completely rule out that changes in MTSS1 expression were a secondary effect which indicated favorable versus poor survival. However, besides being a good prognostic biomarker, it is possible that downregulation of MTSS1 renders cells more resistant to chemotherapy and that alternative treatment strategies are needed for AML with low MTSS1 expression.

One criticism of the present study could be that FLT3-ITD positivity was responsible for the low expression of MTSS1, the high expression of DNMT3B, and the poor survival in normal karyotype-AML cases. Unfortunately, data on the FLT3-ITD status were missing in the analysis by Metzeler et al [19]. However, several lines of evidence point to a more general association of MTSS1 and DNMT3B levels with survival: First, in additional analyses of the 183 AML patients described by Ley et al [34] and the Cancer genome Atlas Network (2013) and the 2096 patients described by Haferlach et al [35], high expression of MTSS1 was associated with the t(8;21) and inv(16) AML subsets while MTSS1 expression was low in 7q-deleted cases and t(11q23)-rearranged cases, both of which are associated with a poor prognosis (not shown). Secondly, while analysis of the Flt3-ITD negative patients included in the analysis of Verhaak et al [36] showed a strong trend for decreased survival of DNMT3B high-expressing AML patients (p = 0.05 each for event-free and overall survival), this was not the case for FLT3-ITD positive patients (not shown). Together with the fact that DNMT3B expression was higher in FLT3-ITD-positive vs. FLT3-ITD-negative patients (not shown), this points to FLT3-ITD induced upregulation of DNMT3B or upregulation of DNMT3B by another yet uncharacterized pathway is a poor prognostic factor in normal karyotype AML.

Taken together, our findings suggest not only that MTSS1 expression is increased in AML subsets with a more favorable outcome but its expression and potential function can be increased by pharmacological approaches allowing for the investigation of future potential alternative therapies, although more in depth genetic and pharmacological studies are required to carefully dissect the exact function of this adapter and tumor suppressor molecule.

## Supporting Information

S1 Fig ATRA induces maturation and differentiation in NB4 and PML9 transduced U937 cells. Human AML cell lines (NB4, U937 or U937 transduced with PMT RARα) were treated with either DMSO control or ATRA to induce myeloid differentiation and maturation as assessed by FACS analysis (n = 3).
(PDF)

S2 Fig Effect of ATRA treatment on Mtss1 expression in PML-RARα positive cells. PML-RARα positive U937 and empty vector control U937 cells (U937 PMT control) were treated using ATRA or DMSO as vehicle control and MTSS1 mRNA levels were assessed by qRT-PCR at indicated time points.
(PDF)

S3 Fig ATRA treatment does not affect Mtss1 expression in FLT3-ITD positive AML. Human FLT3-ITD AML cell line MV4;11 was treated with ATRA and MTSS1 mRNA levels were assessed.
(PDF)
