# Supplementary material for: Identification of the Adapter Molecule MTSS1 as a Potential Oncogene-Specific Tumor Suppressor in Acute Myeloid Leukemia
Source: PLoS One. 2015 May 21;10(5):e0125783. doi: 10.1371/journal.pone.0125783 (PMC4440712; doi:10.1371/journal.pone.0125783)
Supplement: S1 Fig — Human AML cell lines (NB4, U937 or U937 transduced with PMT RARα) were treated with either DMSO control or ATRA to induce myeloid differentiation and maturation as assessed by FACS analysis (n = 3). (PDF) [file pone.0125783.s001.pdf]

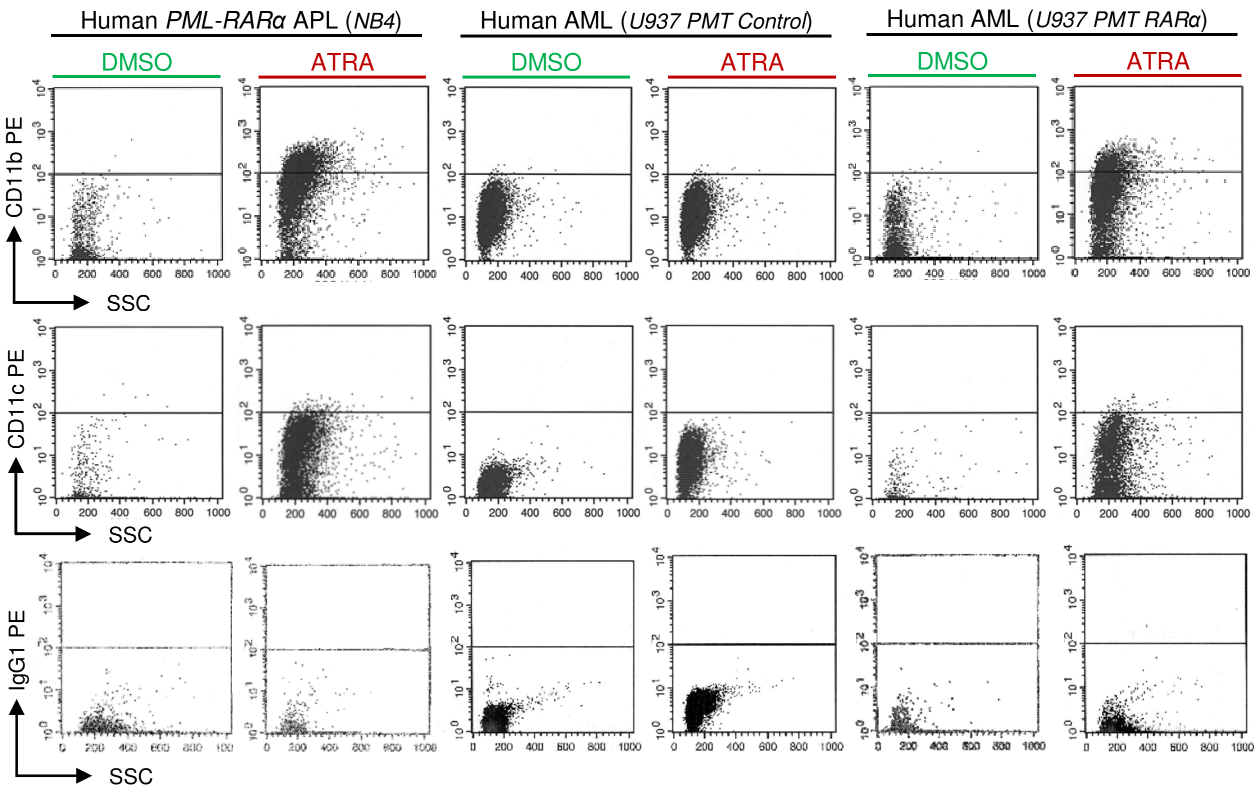

Supporting Information S1. ATRA induces maturation and differentiation in NB4 and PML9 transduced U937 cells. Human AML cell lines (NB4, U937 or U937 transduced with PMT RARα) were treated with either DMSO control or ATRA to induce myeloid differentiation and maturation as assessed by FACS analysis (n=3).
