# Supplementary material for: Identification of the Adapter Molecule MTSS1 as a Potential Oncogene-Specific Tumor Suppressor in Acute Myeloid Leukemia
Source: PLoS One. 2015 May 21;10(5):e0125783. doi: 10.1371/journal.pone.0125783 (PMC4440712; doi:10.1371/journal.pone.0125783)
Supplement: S2 Fig — PML-RARα positive U937 and empty vector control U937 cells (U937 PMT control) were treated using ATRA or DMSO as vehicle control and MTSS1 mRNA levels were assessed by qRT-PCR at indicated time points. (PDF) [file pone.0125783.s002.pdf]

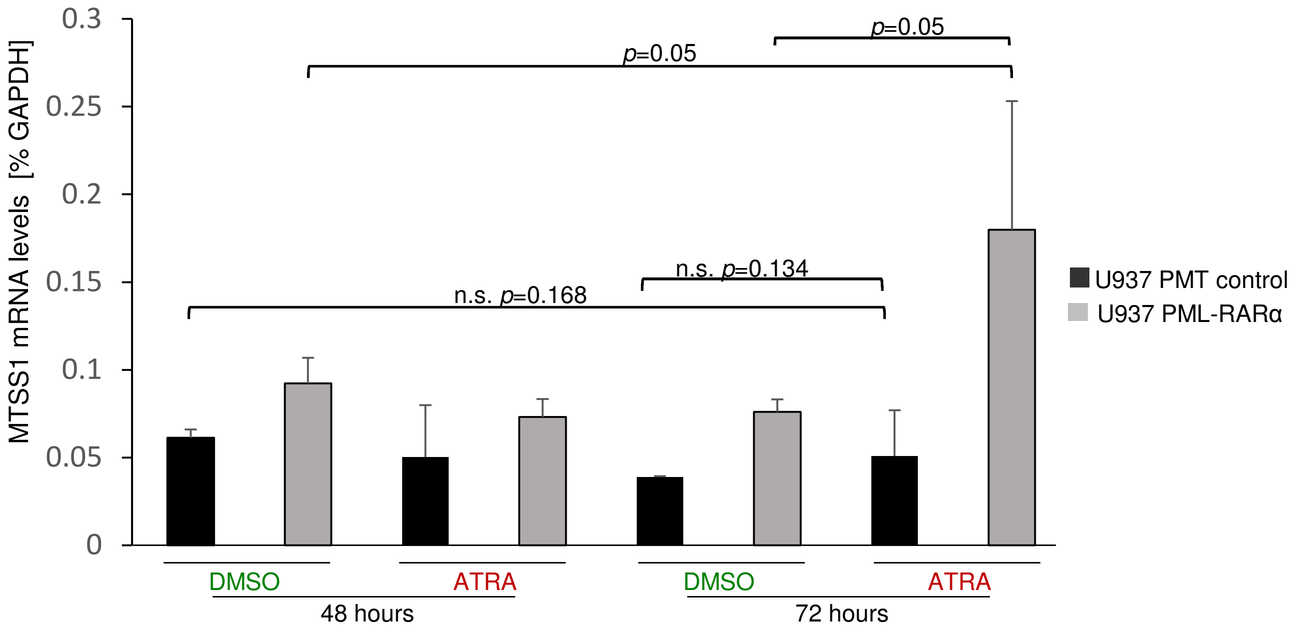

Supporting Information S3. Effekt of ATRA treatment on Mtss1 expression in PML-RARα positive cells. PML-RARα positive U937 and empty vector control U937 cells (U937 PMT control) were treated using ATRA or DMSO as vehicle control and MTSS1 mRNA levels were assessed by qRT-PCR at indicated time points.
