# Supplementary material for: Identification of the Adapter Molecule MTSS1 as a Potential Oncogene-Specific Tumor Suppressor in Acute Myeloid Leukemia
Source: PLoS One. 2015 May 21;10(5):e0125783. doi: 10.1371/journal.pone.0125783 (PMC4440712; doi:10.1371/journal.pone.0125783)
Supplement: S3 Fig — Human FLT3-ITD AML cell line MV4;11 was treated with ATRA and MTSS1 mRNA levels were assessed. (PDF) [file pone.0125783.s003.pdf]

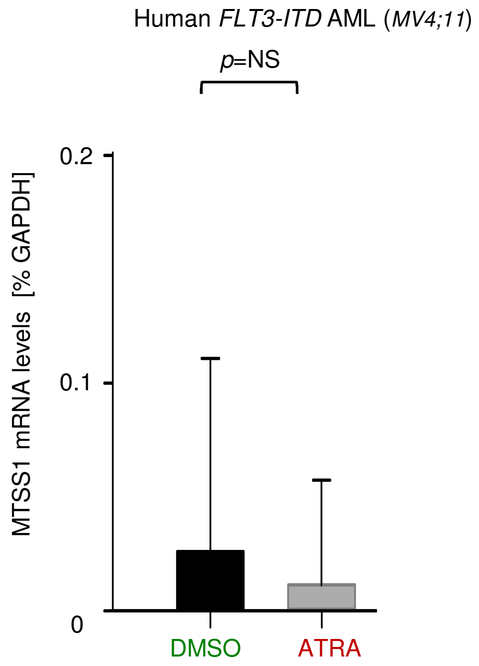


**Supporting Information S2. ATRA treatment does not affect Mtss1 expression in FLT3-ITD positive AML.** Human FLT3-ITD AML cell line MV4;11 was treated with ATRA and MTSS1 mRNA levels were assessed.
